# A khorasan wheat-based replacement diet improves risk profile of patients with type 2 diabetes mellitus (T2DM): a randomized crossover trial

**DOI:** 10.1007/s00394-016-1168-2

**Published:** 2016-02-08

**Authors:** Anne Whittaker, Monica Dinu, Francesca Cesari, Anna Maria Gori, Claudia Fiorillo, Matteo Becatti, Alessandro Casini, Rossella Marcucci, Stefano Benedettelli, Francesco Sofi

**Affiliations:** 1 Department of Agrifood Production and Environmental Sciences, University of Florence, Florence, Italy; 2 Department of Experimental and Clinical Medicine, University of Florence, Florence, Italy; 3 Unit of Atherothrombotic Diseases, Careggi University Hospital, Florence, Italy; 4 Department of Clinical and Experimental Biomedical Sciences, University of Florence, Florence, Italy; 5 Unit of Clinical Nutrition, Careggi University Hospital, Florence, Italy; 6 Don Carlo Gnocchi Foundation Italy, Onlus IRCCS, Florence, Italy

**Keywords:** Organic wheat, Ancient wheat, Modern wheat, Khorasan wheat, Type 2 diabetes mellitus, Secondary prevention, Glycaemia, Oxidative stress, Inflammation cytokines

## Abstract

**Purposes:**

The aim of the present study was to examine whether a replacement diet with products made with organic ancient khorasan wheat could provide additive protective effects in reducing glucose, insulin, lipid and inflammatory risk factors, and in restoring blood redox balance in type 2 diabetes mellitus (T2DM) patients compared to diet with product made with modern organic wheat.

**Methods:**

We conducted a randomized, double-blinded crossover trial with two intervention phases on 21 T2DM patients (14 females, 7 males). The participants were assigned to consume products (bread, pasta, crackers and biscuits) made using semi-whole flour from organic wheat that was either from ancient khorasan wheat or modern control wheat for 8 weeks in a random order. An 8-week washout period was implemented between the interventions. Laboratory analyses were performed both at the beginning and at the end of each intervention phase.

**Results:**

The metabolic risk profile improved only after the khorasan intervention period, as measured by a reduction in total and LDL cholesterol (mean reduction: −3.7 and −3.4 %, respectively), insulin (−16.3 %) and blood glucose (−9.1 %). Similarly, there was a significant reduction in circulating levels of reactive oxygen species (ROS), vascular endothelial growth factor (VEGF) and interleukin-1ra, and a significant increase of total antioxidant capacity (+6.3 %). No significant differences from baseline were noted after the modern control wheat intervention phase. The change (from pre- to post-intervention) between the two intervention arms was significantly different (*p* < 0.05) for total and LDL-c, insulin and HOMA index.

**Conclusions:**

A replacement diet with ancient khorasan wheat consumption provided additive protection in reducing total and LDL cholesterol, insulin, blood glucose, ROS production, and some inflammatory risk factors, which are all key factors warranting of control in secondary prevention of T2DM compared to a diet with products made with modern wheat.

## Introduction

Type 2 diabetes mellitus (T2DM) is a chronic condition characterized by excessive glucose levels, resulting from insulin resistance and/or decreased insulin secretion. T2DM has risen to epidemic proportions worldwide and is estimated to increase even further [1, 2]. A major challenge to the public health sector resides in the treatment of T2DM-derived micro- and macrovascular complications [1, 3]. This highlights the importance of secondary prevention strategies aimed at minimizing endothelial cell dysfunction in T2DM patients, which is the underlying factor in vascular complications [1]. Hyperglycemia results in the generation of reactive oxygen species (ROS) and in the stimulation of inflammatory marker cascades, which induce both endothelial cell dysfunction and insulin resistance, which in turn enhance further hyperglycemia, ROS production and inflammation [1, 3]. Hence, lifestyle interventions aimed at reducing hyperglycemia, ROS production and inflammation in T2DM patients are warranting of consideration. One such lifestyle intervention relates to the role of diet.

Initially, limited experimental evidence has been reported on the effectiveness of diet in secondary prevention for diabetic patients [4]. However, in recent years, there has been increased impetus in investigating the role of diet in T2DM management. In the most recent systematic review with meta-analysis [2], the Mediterranean diet was shown to be associated with better glycemic control and improved cardiovascular risk than control diets.

Given that the present study is based in Italy, the proposed importance of the Mediterranean diet in T2DM management is of interest. Of particular interest is the cereal component, which forms the basis of the Mediterranean dietary pyramid. Although carbohydrates (also derived from cereals) are generally viewed with some degree of trepidation by diabetics, the long-term risks of low carbohydrate diets (usually combined with higher protein intake) are well-described and include mineral, vitamin and fiber deficiencies, and increased cardiovascular risk and related mortality [5]. However, the above-mentioned beneficial effects attributable to carbohydrates are dependent on the source (wholegrain cereals and not those with added fats, sugar and sodium) [5, 6]. Although evidence is inconclusive for an ideal amount of carbohydrate intake for people with diabetes [6], the emphasis is on the quality of the source. Of particular interest are the positive benefits on human health by ancient *Triticum* varieties and *Triticum turgidum* [7, 8] which could serve as a major carbohydrate source in the diet. In our previous study [9], a replacement diet with ancient khorasan wheat products on patients with acute coronary syndrome was shown to result in a significant decrease in lipid and glucose profiles, generation of ROS and lipid peroxidation, as well as in some proinflammatory cytokines. These positive effects were conferred, irrespective of the number and combination of medicinal therapies with proven efficacy in secondary prevention.

Given that T2DM patients have an increased cardiovascular risk, and both chronic diseases share common risk factors, the aim of the present study was to investigate whether a replacement diet with products made from ancient khorasan wheat products could provide additive protective effects on glycemic control, lipid, oxidative and inflammatory risk factors in T2DM patients.

## Materials and methods

### Study population

The study population was initially composed of 24 T2DM patients. Two patients, due to personal reasons (completely unrelated to the study), were unable to participate in the second phase of the project, and a further patient complained of self-diagnosed wheat-related complications during the first phase (notwithstanding the written consent provided at the outset). These three patients were, therefore, excluded from the study. The final study population was comprised of 21 T2DM patients (14 men; 7 women). The mean age was 64.4 ± 10.9 with a mean body mass index (BMI) of 27.9 ± 4.7 (Table 1). All patients were recruited on entry to the clinic following consultation at the Unit of Clinical Nutrition of the Department of Experimental and Clinical Medicine, University of Florence, Careggi University Hospital.

**Table 1:** Baseline characteristics of the study population

Characteristic	Total (*n* = 21)
Age, years (mean ± SD)	64.4 ± 10.9
Sex (M/F)	14/7
BMI (mean ± SD)	27.9 ± 4.7
Duration of T2DM, years (mean ± SD)	3.6 ± 2.2
Hypertension [*n* (%)]	8 (38)
Dyslipidemia [*n* (%)]	7 (33)
Current smokers [*n* (%)]	5 (24)
Sedentary lifestyle [*n* (%)]	13 (62)
MDS (mean ± SD)	10.3 ± 1.6
Cereals’ consumption (1.0–1.5 serving/day, %)	5 (24)
Cereals’ consumption (1.5 serving/day, %)	16 (76)

*BMI* body mass index, *T2DM* type 2 diabetes mellitus, *MDS* Mediterranean diet score, *SD* standard deviation

T2DM was confirmed if at least one or more of the following were reported: (a) HbAc1 ≥6.5 %, (b) fasting plasma glucose ≥126 mg/dL (7.0 mmol/L), (c) 2-h plasma glucose ≥200 mg/dL (11.1 mmol/L) during an oral glucose tolerance test, and (d) a random plasma glucose ≥200 mg/dL (11.1 mmol/L) in patients with symptoms of hyperglycemia. The above-mentioned criteria were consistent with current criteria for the diagnosis of T2DM, according to the American Diabetes Association (2014). Exclusion criteria included clinical manifestations of microvascular complications (retinopathy, neuropathy and nephropathy), macrovascular complications (cardiovascular disease), inflammatory bowel disease, and excess alcohol intake, celiac disease, gluten-sensitivity and wheat allergies.

Patients were instructed not to alter their dietary or lifestyle habits, and written informed consent was then obtained from each participant before the start of the trial. The institutional review board at the University of Florence approved the study protocol.

### Data collection and measurements

Patients underwent an interview, according to standardized methods, to obtain information about personal medical history, demographics, medication, and lifestyle habits. All information relating to the above-mentioned aspects served as descriptive supplementary information pertaining to the current study population, but was not used as a basis for patient exclusion.

Physicians, using standardized protocols, conducted a physical examination, blood pressure measurements, laboratory tests and a dietary survey. BMI was calculated as weight (kg)/height (m^2^). Participants that were smoking at the time of the physical examination were registered as smokers. If physical activity over the preceding 6 months did not meet certain criteria (based on duration and intensity), patients were then documented as having a sedentary lifestyle. Hypertension (raised blood pressure) was defined as systolic blood pressure 140 mmHg or more and/or diastolic blood pressure 90 mmHg, in accordance with the guidelines of the European Society of Cardiology. Dyslipidemia was defined according to the Third Report of the National Cholesterol Education Program (NCEP-III), or if patients reported taking anti-dyslipidemic drugs, as verified by the physician. Adherence to a Mediterranean diet was evaluated from a questionnaire that included 3 categories of consumption (min 0 points, max 2 points) for each food group (cereals, fruit, vegetables, legumes, olive oil, meat products, dairy products, fish and alcohol) representing the major constituents of the Mediterranean diet. The questionnaire permitted the assignment of an overall score (min 0 points, max 18 points) to each patient, thereby ranking the degree of adherence [10].

### Experimental and control wheat

The ancient experimental wheat utilized in the present study was organic KAMUT^®^ khorasan wheat (*Tritucum turgidum* subsp. *turanicum*), provided by Kamut Enterprises of Europe (KEE), Belgium (from here on referred to as the khorasan wheat). KAMUT^®^ is a registered trademark of Kamut International, Ltd. and Kamut Enterprises of Europe, bvba and guarantees the wheat is pure ancient khorasan wheat and is organically grown and processed. As the control (from here on referred to as the control wheat), a mix of organic modern commercial Italian durum (*T. durum*) varieties and soft wheat (*T. aestivum*) varieties, respectively, were used. Various reported biochemical parameters of the khorasan and control semolina and flour (Table 2) were determined as previously [8]. Given that different countries utilize different terminology to classify the different types of milled flour, we report the ash content (positively correlated to the extraction rate) for comparative purposes. Khorasan semolina (ash content 1.10–1.35 %) and flour (ash content 1.0 %) were processed by Molino SIMA S.C.A.R.L. (Argenta, Ferrara, Italy). For the organic khorasan wheat, different milling procedures were employed to produce the granulated semolina (analogous to semi-whole wheat semolina) and flour (analogous to semi-whole wheat flour), respectively, thereby resulting in differences in ash content. For the control, semi-whole-wheat granulated semolina (ash content 1.0–1.35 %) and semi-whole wheat flour (ash content 0.95 %), respectively, were similarly processed by Molino SIMA.

**Table 2:** Composition of Kamut and control wheat (for 100 g)

Variable	Khorasan semolina	Control semolina	*p* value	Khorasan flour	Control flour	*p* value
Total protein (%)	14.3 ± 0.6	14.3 ± 0.4	0.8	14.7 ± 0.1	10.3 ± 0.08	<0.05
Total starch (%)	70.4 ± 1.2	68.6 ± 1.2	0.3	69.4 ± 2.3	70.5 ± 0.3	0.5
Iron (mg/kg)	28.5 ± 1.2	31.0 ± 4.0	0.5	23.5 ± 0.5	22.8 ± 0.5	0.1
Magnesium (mg/kg)	957.4 ± 12.3	789.7 ± 115.3	<0.05	803.6 ± 5.1	673.1 ± 109.2	<0.05
Manganese (mg/kg)	21.1 ± 1.1	11.1 ± 0.4	<0.05	14.3 ± 0.6	15.5 ± 1.3	0.1
Phosphorus (mg/kg)	2841.5 ± 94.5	2432.0 ± 112.8	<0.05	2314.8 ± 76.7	1613.3 ± 41.3	<0.05
Potassium (mg/kg)	2404.4 ± 46.6	1969.8 ± 10.2	<0.05	2093.0 ± 4.0	1531.6 ± 17.1	<0.05
Zinc (mg/kg)	25.5 ± 0.2	25.3 ± 0.2	0.1	19.9 ± 0.4	18.3 ± 0.5	<0.05
Selenium (mg/kg)	1.0 ± 0.04	0.9 ± 0.03	0.2	0.9 ± 0.01	0.7 ± 0.01	<0.05
Total polyphenols (mg/g DM)	2.0 ± 0.2	1.8 ± 0.1	<0.05	1.8 ± 0.1	1.4 ± 0.2	<0.05
ARP (antiradical power)	6.3 ± 0.5	5.9 ± 0.7	0.5	6.3 ± 0.6	4.8 ± 0.2	<0.05

Data are reported as mean and standard deviation. One-way ANOVA test

Pastificio Artigiano FABBRI s.a.s. (Strada in Chianti, Firenze, Italy) prepared the pasta (with no additives) from both the khorasan and control semolina, according to the artisan manufacturing procedures. The artisan enterprise of Panificio Menchetti Pietro di Santi e Figli s.n.c. (Cesa Marciano della Chiana, Arezzo, Italy) prepared the bread, biscuits and crackers using the khorasan and control flour. Naturally, leavened Tuscan-style sourdough bread was prepared. Besides the flour composition, dry crackers contained 20 % extra-virgin olive oil. The biscuits were prepared using 10 % sugar, 5 % butter, and one egg per 100 g flour.

### Study design

The study was a randomized, double-blinded, crossover trial aimed at testing whether a replacement diet with khorasan wheat products and/or control wheat products could provide additive benefits to T2DM patients. For this reason, other cereals were excluded from the diet and “replaced” by either the khorasan or control products during the respective intervention phases. The food products were packaged with no labels attached to the packages, and patients were informed that all products to be administered were organic and prepared by artisan methods. The patients were randomly divided into two groups (Group A and Group B), and a crossover study design with two intervention phases was implemented. The first intervention phase was initiated in late January 2015, with Group A and B, respectively, initiating the trial with organic khorasan and control products. Participants in both groups received 500 g/week of pasta, 150 g/day of bread, 250 g/week of crackers, and 250 g/week of biscuits for a period of 8 weeks. Patients were advised to eat the products according to their normal cereal consumption habits, which were documented at baseline. A washout period of 8 weeks was then affected, during which patients were permitted to eat all foods according to their “normal” dietary habits. The second intervention phase of 8 weeks was initiated in early May 2015, and Group A and B crossed-over to consume the control and khorasan products, respectively. On average, patient’s daily intake of both khorasan and control semolina was 62 g dry weight, whereas daily intake of khorasan or control flour (from all the products consumed) amounted to 140 g dry weight. The energy (kcal) provided by semolina and flour was 221 and 501 kcal, respectively, making a total of 722 kcal (50–55 % of daily energy intake) replaced in this intervention. At baseline and after each intervention, all subjects were examined between 7:00 a.m. and 9:30 a.m. after an overnight fasting period. Furthermore, subjects were asked not to engage in strenuous physical activity during the day before the examination.

### Laboratory measurements

Venous blood samples were collected into evacuated plastic tubes (Vacutainer). Samples were centrifuged at 3000*g* for 15 min (4 °C) and stored in aliquots at −80 °C until further analysis. Cholesterol subtypes, triglycerides, glucose, insulin, HbA1c, and serum electrolytes were assessed according to conventional methods. Pro- and anti-inflammatory cytokines were determined by using the Bio-Plex cytokine assay (Bio-Rad Laboratories Inc., Hercules, CA, USA), according to the manufacturer’s instructions.

### Assessment of ROS production and total antioxidant capacity

Leukocyte (lymphocyte, monocyte and granulocyte) ROS generation was measured as reported previously [8, 9]. Similarly, fatty acid peroxidation was determined by measuring malondialdehyde (MDA) using the thiobarbituric acid reactive substance (TBARS) assay kit (Oxitek-ZeptoMetrix Corporation Buffalo, NY, USA). Total antioxidant capacity (TAC), accounting for total hydrophilic ROS scavengers, was measured using the ORAC assay (oxygen radical absorbance capacity), based on the inhibition of the peroxyl-radical-induced oxidation initiated by thermal decomposition of azo-compounds, like 2,2-azobis (2-amidinopropane) dihydrochloride (AAPH), was performed as previously described [11].

### Statistical analysis

The statistical package PASW 20.0 for Macintosh (SPSS Inc., Chicago, IL, USA) was utilized. Results were expressed either as mean ± SD or as median and range. One-way ANOVA was used for testing differences between khorasan and control flour and semolina. The analyses were simplified by calculating the absolute change for each variable tested (mean value at baseline subtracted from the mean value after intervention for each subject) with independent t sample tests. All data were treated as paired samples from a crossover study. Data that were not normally distributed were logarithmically transformed. The two interventions were analyzed by taking into account both phases in the two groups of subjects at different stages. Data were analyzed by using paired t tests for significant differences between changes observed during test and control intervention periods. Moreover, in order to compare the effect of organic khorasan products versus baseline and versus the control products, a general linear model for repeated measurements, after adjustment for age and gender, modifiable risk factors, diet quality, and antidiabetic medication was performed. A value of *p* ≤ 0.05 was considered to indicate statistical significance.

## Results

### Study population

Baseline characteristics of the study population, including time since the onset of T2DM, traditional cardiovascular risk factors, lifestyle habits, medications, and diet quality are presented in Table 1. Nearly two-thirds of the population was sedentary, and the vast majority consumed 1.5 portions (200 g) of cereals a day. All the patients were under pharmacological treatment for T2DM: 8 (38 %) took metformin, 9 (43 %) hypoglycemic, and 4 (19 %) DPP-4. The patients reported not having changed either medication or lifestyle habits (smoking, physical activity, adherence to Mediterranean diet) during the course of the study. At the end of the study, there were no significant differences (*p* > 0.05) in body weight change between khorasan and control arms (−0.3 ± 1.8 and −0.6 ± 1.2 kg, respectively).

### Wheat characteristics

As reported in Table 2, total polyphenol content and antiradical power (ARP) were significantly higher in the khorasan flour in comparison with the control flour. Magnesium, potassium and phosphorus were significantly higher in both the khorasan semolina and flour compared to that of the control. Higher levels of manganese and polyphenols were recorded the khorasan semolina than in the control semolina, whereas higher levels of selenium and protein were recorded in the khorasan flour compared to the control.

### Modifications in lipid and metabolic profiles

In order to evaluate the differences in change from pre- to post-intervention between khorasan and control groups, we applied a general linear model adjusted for age, gender, BMI, hypertension, diet score, and anti-diabetic drugs. There were significant differences between study arms regarding the absolute changes in total and LDL cholesterol, insulin, and HOMA index. As reported in Table 3, serum levels of total cholesterol (−3.7 %), LDL cholesterol (−3.4 %), blood glucose (−9.1 %) and insulin (−16.3 %) significantly improved after the consumption of khorasan products. On the other hand, no significant effect was noted after the consumption of the control diet. Finally, we observed a trend for a significant increase of magnesium after the period of intervention with khorasan wheat, but the result did not reach the statistical significance when fully adjusted model was applied.

**Table 3:** Modification of biochemical parameters

Variable	Khorasan pre	Khorasan post	*p* value^†^	Control pre	Control post	*p* value^†^	Change khorasan	Change control	*p* value^†^
Cholesterol (mg/dL)	189.8 ± 38.9	182.8 ± 38.7	0.047	183.8 ± 35.8	189.5 ± 37.5	0.167	−7.00 (−13.9; 0.09)	5.71 (−2.71; 14.1)°	0.012
LDL cholesterol (mg/dL)	116.6 ± 33.3	107.8 ± 34.9*	0.049	114.2 ± 31.7	118.5 ± 30.9	0.319	−8.81 (−17.56; 0.06)	4.26 (−4.73; 13.3)°	0.012
HDL cholesterol (mg/dL)	45.9 ± 10.5	45.3 ± 11	0.580	46 ± 14.4	46.2 ± 9.3	0.902	−0.62 (−2.98; 1.74)	0.19 (−3.10; 3.48)	0.754
Triglycerides (mg/dL)	155.5 ± 72	157.3 ± 70	0.862	148.1 ± 61.8	164.5 ± 78.3	0.173	1.81 (−20.2; 23.8)	16.4 (−8.19; 41.0)	0.402
Glycemia (g/L)	1.53 ± 0.66	1.39 ± 0.57*	0.049	1.39 ± 0.50	1.44 ± 0.65	0.488	−0.14 (−0.28; 0.001)	0.06 (−0.12; 0.24)	0.076
Insulin (mU/L)	14.7 ± 9.2	12.3 ± 7.6*	0.045	11.8 ± 6.4	13.4 ± 7.5	0.087	−2.44 (−4.81; −0.06)	1.56 (−0.26; 3.37)°	0.011
HOMA index	5.52 ± 3.8	4.18 ± 3.31*	0.054	3.89 ± 2.38	4.58 ± 2.94	0.089	−1.34 (−2.72; 0.03)	0.69 (−0.12; 1.50)°	0.036
HbAc1 (%)	57.3 ± 22.2	57.2 ± 18.9	0.990	56 ± 22.8	55.6 ± 21.6	0.647	−0.01 (−2.47; 2.44)	−0.46 (−2.57; 1.65)	0.791
Potassium (mEq/L)	4.24 ± 0.37	4.19 ± 0.43	0.447	4.36 ± 0.44	4.25 ± 0.41	0.164	−0.06 (−0.21; 0.1)	−0.11 (−0.27; 0.05)	0.615
Calcium (mEq/L)	9.05 ± 0.27	9.03 ± 0.25	0.827	9.07 ± 0.37	9.18 ± 0.30	0.306	−0.02 (−0.20; 0.17)	0.11 (−0.11; 0.33)	0.362
Magnesium (mg/dL)	1.8 ± 0.19	1.9 ± 0.27*	0.083	1.8 ± 0.18	1.8 ± 0.25	0.474	0.07 (−0.01; 0.15)	0.03 (−0.06; 0.11)	0.407
Iron (μg/dL)	83.7 ± 29.1	85.4 ± 28.7	0.801	75.1 ± 22.5	89.1 ± 27.1*	0.023	1.71 (−12.7; 16.1)	13.9 (2.22; 25.7)	0.150

Data are reported as mean and standard deviation

* *p* < 0.05 for paired *T* test (pre vs post)

° *p* < 0.05 for independent *T* test (for changes between the khorasan and control groups)

^†^General linear model for repeated measurements adjusted for age, gender, BMI, hypertension, diet score, and anti-diabetic drugs

### Modifications in blood redox status

Blood redox status, before and after each respective intervention phase, was tested (Table 4). The difference for changes in TAC between the two study arms was statistically significant, as well as for granulocytes-derived ROS. TAC was significantly increased after the consumption of the khorasan wheat products and significantly decreased after the consumption of modern wheat products, whereas ROS production in both circulating granulocytes and monocytes was significantly reduced after the khorasan wheat replacement diet. No effect was evident after the consumption of control wheat products. Lymphocyte ROS production and the plasma levels of MDA remained unchanged after both the khorasan wheat and control dietary interventions.

**Table 4:** Modification of oxidative stress parameters

Variable	Khorasan pre	Khorasan post	*p* value^†^	Control pre	Control post	*p* value^†^	Change khorasan	Change control	*p* value^†^
TBARS (nmol/mL)	40.9 ± 14	38.3 ± 13.5	0.163	43.8 ± 13.2	45.5 ± 13.1	0.390	−2.58 (−6.03; 0.88)	1.71 (−2.44; 5.85)	0.157
L-derived ROS, RFU	1320.8 ± 294.5	1149.6 ± 473.1	0.180	1260.3 ± 256.8	1264.7 ± 410.3	0.964	−171.3 (−432.1; 89.6)	4.35 (−201.3; 209.9)	0.273
M-derived ROS, RFU	2509.4 ± 455.4	1890.7 ± 703.7*	0.002	2452.8 ± 545.9	2285 ± 610.3	0.349	−618.7 (−972.8; −264.7)	−167.8 (−541; 205.3)	0.092
G-derived ROS, RFU	3067.9 ± 606.3	2503.4 ± 809.9*	0.005	2846.3 ± 824.5	2916.2 ± 728.4	0.747	−564.4 (−921.1; −207.8)	69.9 (−388.1; 527.9)°	0.025
TAC (μmol/mL)	20.8 ± 3.6	22.3 ± 3.3*	0.049	21.8 ± 2.2	20.5 ± 2.0*	0.024	1.41 (0.01; 2.08)	−1.34 (−2.47; −0.21)°	0.017

Data are reported as mean and standard deviation

*L* lymphocytes, *M* monocytes, *G* granulocytes, *ROS* reactive oxygen species, *RFU* relative fluorescence unit, *TAC* total antioxidant capacity

* *p* < 0.05 for paired *T* test (pre vs post)

° *p* < 0.05 for independent *T* test (for changes between the khorasan and control groups)

^†^General linear model for repeated measurements adjusted for age, gender, BMI, hypertension, diet score, and anti-diabetic drugs

### Modifications in the inflammatory profile

After adjustment for age, gender, BMI, hypertension, diet score, and anti-diabetic drugs, significant decreases in only VEGF and IL-1ra were evident following the organic khorasan replacement diet (Table 5). A slight but significant increase in IL-8 was evident after consumption of the control. No significant changes were reported for the remaining pro- and anti-inflammatory cytokines.

**Table 5:** Modifications in the inflammatory profile

Variable	Khorasan pre	Khorasan post	*p* value^†^	Control pre	Control post	*p* value^†^	Change khorasan	Change control	*p* value^†^
Interleukin-1ra (pg/mL)	38.9 ± 23.2	28.3 ± 24.3*	0.004	27.5 ± 23.5	30.3 ± 28.1*	0.555	−10.6 (−17.2; −4.1)	2.76 (−7.04; 12.6)°	0.008
Interleukin-4 (pg/mL)	0.43 ± 0.38	0.55 ± 0.41	0.352	0.45 ± 0.40	0.39 ± 0.37*	0.502	0.11 (−0.14; 0.37)	−0.06 (−0.23; 0.12)	0.277
Interleukin-6 (pg/mL)	2.76 ± 2.01	2.16 ± 1.21	0.276	2.15 ± 1.57	1.70 ± 1.24	0.09	−0.59 (−1.72; 0.53)	−0.44 (−0.96; 0.08)	0.698
Interleukin-8 (pg/mL)	5.39 ± 3.12	6.75 ± 2.43	0.069	3.75 ± 1.72	6.52 ± 3.04	0.001	1.36 (−0.12; 2.83)	2.78 (1.32; 4.24)	0.229
Interleukin-10 (pg/mL)	10.3 ± 5.6	10.9 ± 7.9*	0.631	10.8 ± 7.5	10.1 ± 5.6	0.737	0.69 (−2.35; 3.75)	−0.63 (−4.57; 3.31)	0.664
Interleukin-12 (pg/mL)	29.1 ± 23.2	31.4 ± 26.5*	0.538	30.3 ± 21.6	26.3 ± 17.9*	0.516	2.27 (−5.44; 9.97)	−3.97 (−16.8; 8.82)	0.404
Interleukin-17 (pg/mL)	7.47 ± 3.8	7.07 ± 2.94	0.677	5.48 ± 3.42	7.06 ± 3.69	0.161	−0.40 (−2.41; 1.61)	1.58 (−0.71; 3.88)	0.181
INF-gamma (pg/mL)	22.2 ± 15	21.5 ± 18.1	0.884	40.9 ± 14	38.3 ± 13.5*	0.141	−0.74 (−11.5; 10)	−6.44 (−15.3; 2.4)	0.240
MCP-1 (pg/mL)	57.8 ± 39.4	50.5 ± 31.9*	0.320	43.1 ± 26.7	50.4 ± 34.5*	0.217	−7.27 (−22.4; 7.86)	7.37 (−4.86; 19.6)	0.109
MIP-1 beta (pg/mL)	62.7 ± 31	68.7 ± 35.6*	0.394	67.6 ± 41	66.8 ± 35*	0.921	6.01 (−8.66; 20.7)	−0.85 (−19.1; 17.4)	0.631
TNF-alpha (pg/mL)	4.54 ± 3.32	4.74 ± 3.09	0.776	4.36 ± 4.09	4.84 ± 4.07*	0.643	0.20 (−1.28; 1.68)	0.48 (−1.69; 2.64)	0.798
VEGF (pg/mL)	189.5 ± 165.5	125.8 ± 114.9*	0.027	144.5 ± 133.4	128.1 ± 127.8*	0.364	−63.7 (−119.2; −8.24)	−16.4 (−53.9; 21.1)	0.113

Data are reported as mean and standard deviation

*INF*-*gamma* interferon-gamma, *MCP*-*1* monocyte chemotactic protein-1, *MIP*-*1beta* macrophage inflammatory protein-1 beta, *TNF*-*alpha* tumor necrosis factor-alpha, *VEGF* vascular endothelial growth factor

* *p* < 0.05 for paired *T* test (pre vs post)

° *p* < 0.05 for independent *T* test (for changes between the khorasan and control groups)

^†^General linear model for repeated measurements adjusted for age, gender, BMI, hypertension, diet score, and anti-diabetic drugs

## Discussion

The present work is the second study that evaluated the functional efficacy of organic ancient khorasan wheat in a population with a chronic disease, notwithstanding the use of drug therapy. The incentive to investigate the effect of an organic ancient khorasan wheat replacement diet on T2DM patients was based on the positive benefits shown in our previous study on ACS patients [9]. In the current study, all patients were on effective glucose lowering therapies, with positive albeit variable medication-dependent pleiotropic effects reported for cholesterol and inflammatory markers [12, 13]. Given that medicinal therapy and lifestyle habits (smoking and sedentary lifestyle/physical exercise, Mediterranean diet adherence) were maintained constant throughout the trial, the study permitted the evaluation of beneficial changes attributable to a replacement diet with either the organic ancient khorasan or the organic modern control wheat. Here, we report a positive impact of khorasan wheat products on blood insulin and glucose. With regard to insulin resistance (HOMA index), a reduction after the khorasan phase was observed. Moreover, the absolute change between pre- and post-intervention was statistically significant when compared with the change in the control group. Nevertheless, the imbalance between the 2 intervention groups in baseline values could bias this result.

Secondary prevention improvements, important in reducing the potential risk of vascular complications in T2DM patients, were also evident from the significant decrease in LDL cholesterol, granulocyte and monocyte ROS production (with a simultaneous improvement in plasma TAC), and proinflammatory IL-1ra and VEGF. Similar benefits were not evident after the consumption of the commercial modern wheat control. Given that T2DM patients have a considerably higher risk of cardiovascular morbidity and mortality [14] and LDL is the predominant cholesterol risk factor, successful dietary/lifestyle treatments aimed at reducing this parameter are warranting of attention in T2DM secondary prevention.

The present study further corroborates previous research from our group [8, 9, 15], demonstrating the health-promoting benefits of organic ancient khorasan wheat, not evident in the organic modern control wheat. As outlined in previous studies, differences in protective effects were not attributable to the fact that khorasan wheat was organic semi-whole wheat, since the same patients also consumed the organic, semi-whole control wheat. Despite the recent benefits reported on Mediterranean diet (as a whole) in T2DM management [2], the present study demonstrates that the potential for further improvement, just by modifying a single food component within the Mediterranean diet, namely substituting ancient cereals for modern cereals, is worthy of consideration. It is important to highlight that the beneficial effects observed in the present study from the khorasan wheat were on patients with a diagnosis of T2DM for an average of 3.6 years, without vascular complications and who were taking medication singularly. Whether similar benefits could be observed for T2DM patients in tertiary prevention on multiple medication remains to be investigated.

In line with our previous studies [8, 9, 15], khorasan wheat was found to be richer in macro- and microelements, with slight but significantly higher levels of polyphenol secondary metabolites, particularly in comparison with the control modern soft wheat. The beneficial role of polyphenols in T2DM is receiving attention, and these benefits include: protection of pancreatic beta-cells against glucose toxicity, anti-inflammatory (modulation of NF-Kb, activation of TNF-alpha, IL-6 and VEGF) plus antioxidant effects, decreased starch digestion, inhibition of advanced glycation end product formation, and beneficial effects on gut microflora [16].

Control of hyperglycemia is one of the important secondary prevention strategies in T2DM. HbA1c, which is the ADA “gold standard” for glucose management in diabetic patients, remained unchanged. Given that HbA1c is linked to the life span of red blood cells, it is evident that the duration of the present trial was too short to accurately determine HbA1c. On the other hand, notwithstanding effective medicinal therapies taken by all the participants, consumption of khorasan affected a significant decrease in insulin and glucose levels. It has been shown that low-GI diets promote significantly improved lipid profiles in medium- and longer-term interventions, particularly with regard to lowered total cholesterol and LDL cholesterol levels [17–19]. Accordingly, the consumption of khorasan wheat products also resulted in a significant decrease in LDL cholesterol and total cholesterol. Alterations in lipid metabolism in T2DM (associated with IR) do not necessarily affect LDL cholesterol levels, but the risk of LDL oxidation, due to increased ROS and an impaired antioxidant scavenging system, is elevated [5, 12, 14].

A significant decrease in ROS production of circulating granulocytes and monocytes, after a replacement diet with khorasan wheat products, was evident. Moreover, there was a slight, but significant, increase in plasma TAC levels. This effect was not evident after the replacement diet with control products. Both monocytes and neutrophils (main constituent of granulocytes) are reported to be defective in almost all physiological functions of the diabetic patients, and granulocytes have been shown to be particularly vulnerable to oxidative stress damage in T2DM patients, notwithstanding metformin usage [20]. Given that both leukocyte types are sources of proinflammatory cytokines and that oxidative stress in T2DM is the common denominator for the major pathways involved in the development of diabetic complications [1], the efficacy of khorasan wheat in reducing ROS production is noteworthy.

In the present study, proinflammatory VEGF decreased significantly only after the consumption of khorasan wheat products. Circulatory baseline levels of VEGF in the T2DM patients of the present study were similar to those reported by Lim et al. [21] and were significantly higher than the baseline levels of the healthy asymptomatic controls selected in both that study and that of Sofi et al. [8]. Interestingly, in our previous study on ACS patients [9], baseline VEGF levels were similar to those found in the present study, and the consumption of khorasan wheat products was also shown to effect a similar mean decrease, even if significance was not attained due the greater variation in VEGF levels. T2DM-induced hyperglycemia and resultant oxidative stress stimulate the upregulation of VEGF, thereby redirecting the physiological role of VEGF toward that of a pathological nature resulting in excessive angiogenesis [3]. Overexpressed VEGF is a proven causative agent in increased permeability of endothelial cells (leakage), and in regulating subsequent inflammatory responses, resulting in the progression of vascular diabetic complications [3]. Hence, strategies aimed at reducing VEGF levels are considered to have therapeutic significance in the secondary prevention care of diabetes [3]. Hence, the consumption of healthy food products, such as those made from khorasan wheat, may provide some relief as healthy alternative risk management in reducing VEGF levels.

The present study was subjected to limitations. The size of the patient population was small, and the study will have to be conducted on a larger population to verify results. Furthermore, blood pressure variations were not taken into account. However, the advantage was there was a range of response for traditional risk factors for vascular complications in this small T2DM population, and despite this variation, there were overall significant changes in various parameters, which could then be independently attributed to the replacement diet. Body weight did not change significantly after the trial showing that patients adhered to their normal consumption of carbohydrates and that the beneficial effects were not associated with weight loss, but with the type of grain consumed.

In conclusion, a replacement diet with organic ancient khorasan wheat products was effective in improving total and LDL cholesterol, blood insulin, HOMA index, ROS production (with a simultaneous improvement in plasma TAC), and proinflammatory IL-1ra and VEGF. Despite the pleiotropic effects of the medication, organic ancient khorasan consumption within an already beneficial Mediterranean diet provides additive protection in reducing hyperglycemia, ROS production and inflammation in T2DM, which are key areas warranting of control in secondary prevention.
